# Phenotypical Changes of Hematopoietic Stem and Progenitor Cells in Sepsis Patients: Correlation With Immune Status?

**DOI:** 10.3389/fphar.2020.640203

**Published:** 2021-01-19

**Authors:** Ping Wang, Jun Wang, Yi-hao Li, Lan Wang, Hong-cai Shang, Jian-xun Wang

**Affiliations:** ^1^ School of Life Sciences, Beijing University of Chinese Medicine, Beijing, China; ^2^ Department of Critical Care Medicine, Dongzhimen Hospital, Beijing University of Chinese Medicine, Beijing, China; ^3^ Key Laboratory of Chinese Internal Medicine of Ministry of Education and Beijing, Dongzhimen Hospital, Beijing University of Chinese Medicine, Beijing, China

**Keywords:** sepsis, hematopoietic stem cells, progenitor cells, phenotypical changes, immune status

## Abstract

**Background:** Sepsis is life-threatening organ dysfunction associated with high risk of death. The immune response of sepsis is complex and varies over time. The immune cells are derived from hematopoietic stem and progenitor cells (HSPCs) which can respond to many infections. Our previous study found that sepsis causes HSPC dysregulation in mouse. But few studies have previously investigated the kinetics of HSPC and its contribution to immune system in sepsis patients.

**Purpose:** We aimed to identify the kinetics of HSPCs and their contribution to immune system in sepsis patients.

**Methods:** We enrolled eight sepsis patients and five healthy control subjects. Peripheral blood (PB) samples from each patient were collected three times: on the first, fourth, and seventh days, once from each healthy control subject. Peripheral blood mononuclear cells (PBMCs) were isolated by density centrifugation and stained with cocktails of antibodies. Populations of HSPCs and their subpopulation were analyzed by flow cytometry. Immune cells were characterized by flow cytometry and blood cell analysis. Correlations between HSPCs and immune cells were analyzed using the Pearson correlation test.

**Results:** We found that the frequency of HSPCs (CD34^+^ cells and CD34^+^CD38^+^ cells) in sepsis patients on day 4 was significantly higher than that in the healthy controls. The most pronounced change in subpopulation analysis is the frequency of common myeloid progenitors (CMPs; CD34^+^CD38^+^CD135^+^CD45RA^−^). But no difference in the immunophenotypically defined hematopoietic stem cells (HSCs; CD34^+^CD38^−^CD90^+^CD45RA^−^) in sepsis patients was observed due to rare HSC numbers in PB. The number of PBMCs and lymphocytes are decreased, whereas the white blood cell (WBC) and neutrophil counts were increased in sepsis patients. Importantly, we found a negative correlation between CD34^+^ on day 1 and WBC and lymphocytes on day 4 from correlation analysis in sepsis patients.

**Conclusion:** The present study demonstrated that the HSPC and its subpopulation in sepsis patients expanded. Importantly, the changes in HSPCs at early time points in sepsis patients have negative correlations with later immune cells. Our results may provide a novel diagnostic indicator and a new therapeutic approach.

## Introduction

Sepsis is life-threatening organ dysfunction, a devastating consequence of infection with bacteria, viruses, or fungi (Singer et al., 2016). Despite the continuous progress in the treatment of sepsis, no effective therapy is available, and it still remains one of the leading causes of mortality in most intensive care units (Ward and Fattahi, 2019; Rudd et al., 2020). Thus, effective treatment should focus on earlier identification and timely intervention (Cecconi et al., 2018). The pathogenesis of the sepsis syndrome is very complex. The host immune response during sepsis is recognized to involve excessive activation of both pro- and anti-inflammatory responses to infection that is followed by exhaustion of mature immune cells such as neutrophils and lymphocytes (Singer et al., 2016; van der Poll et al., 2017). These cells of the innate and adaptive immune systems involved in pathogenesis are derived from a small number of hematopoietic stem and progenitor cells (HSPCs). Recent studies suggest that HSPCs respond directly and immediately to many infections to replenish the mature immune cells (Pietras et al., 2016; Pietras, 2017; Singh et al., 2018). Therefore, investigating the contribution of HSPCs to the immune system during sepsis may shed new light on the early diagnosis and treatment on this significant health problem.

Hematopoietic stem cells (HSCs) are responsible for production all of blood and immune cells over the life span and normally maintain a quiescent state (Yu and Scadden, 2016). At the same time, they rapidly exit the quiescent state and transiently proliferate in response to acute infection, which is referred to as emergency hematopoiesis (King and Goodell, 2011; Manz and Boettcher, 2014). However, our previous study found that sepsis causes HSPC dysregulation, promotes acute expansion of myeloid progenitors, and results in failure to generate mature hematopoietic cells in the sepsis mouse (Wang et al., 2018). Intriguingly, the research studies regarding HSPCs in sepsis animal models also show that sepsis lead to an expansion and functional impairment of HSPCs (Rodriguez et al., 2009; Skirecki et al., 2015; Zhang et al., 2016). The correlative literature suggests that HSPCs draw particular attention because they may play a foundation role for the immune response during sepsis, and it remains to be evaluated whether impaired hematopoiesis also occurs in sepsis patients.

To date, few studies have previously investigated kinetics of HSPCs and their contribution to the immune system in sepsis patients. Tsaganos et al. found that CD34/CD45^−^–positive cells in the peripheral blood have increased throughout the days of the follow-up compared with healthy volunteers (Tsaganos et al., 2006), whereas Skirecki et al. found that populations of early HSCs (CD34^+^CD38^−^) were mobilized to the peripheral blood after an initial decrease (Skirecki et al., 2019). And both of them indicated that patients with less stem cell counts are accompanied by a greater probability of survival. However, despite CD34 being the most important marker of primitive human hematopoietic cells, and CD34^+^ and CD34^+^CD38^−^ populations both heterogeneous, it does not provide an accurate measure of HSCs and immature progenitors (Pang et al., 2011).

In the present study, we aimed to more precisely identify the kinetics of HSPCs and their contribution to the immune system in sepsis patients. A six-color protocol of antibodies was used to evaluate the frequency and distribution of HSPCs and their subpopulations circulating in blood in sepsis and healthy control subjects by flow cytometry. In addition, correlation analysis was used to analyze the correlation between HSPCs and mature immune cells.

## Materials and Methods

### Subjects

We enrolled eight sepsis patients and five healthy control subjects from Dongzhimen Hospital in Beijing, China (Table 1). The study was conducted according to the protocol which was approved by the institutional ethics committee in accordance with the Helsinki Declaration (Ethics No. DZMEC-KY-2019-101, Supplementary Figure S1). Written informed consent for peripheral blood (PB) collection was obtained from each subject. The diagnostic criteria of sepsis were according to the Third International Consensus Definitions for Sepsis and Septic Shock (Singer et al., 2016). Patients will be excluded if they fulfill any of the exclusion criteria: ages <40 or >80 years, severe hematological diseases, unrespectable tumors, human immunodeficiency virus (HIV), immunosuppressive treatment, and pregnancy. All patients received standard care according to the International Guidelines for Management of Sepsis and Septic Shock (Rhodes et al., 2017).

**TABLE 1:** Comparison of demographic and basal clinical characteristics between sepsis patients and healthy control subjects.

Characteristic	Sepsis (*n* = 8)	Control (*n* = 5)
Age, median (range)	63.5 (42–77)	67 (48–72)
Sex, male/female	5/3	3/2
Hospital mortality, *n* (%)	3 (37.5%)	—
SOFA score, mean ± SEM	5.88 ± 0.64	—
Vital signs, mean ± SEM		
Systolic blood pressure	147.00 ± 12.41	107.80 ± 4.26
Pulse rate	96.25 ± 6.54	86.60 ± 6.31
Respiratory	25.50 ± 3.34	18.40 ± 0.93
Temperature	37.53 ± 0.32	36.36 ± 0.14
Infection source, *n* (%)		
Lung	4 (50%)	—
Heart	3 (38%)	—
Kidney	3 (38%)	—
Liver	2 (25%)	—
Other	2 (25%)	—

SEM, standard error of mean; SOFA, sequential organ failure assessment score.

### Sample Collection and Measurement

EDTA-anticoagulated PB samples (6 ml) were collected three times from each patient: within 24 h after included, on day 4, and on day 7, once from each healthy control subject. Laboratory assessment including measurement of the full blood count and flow cytometric analysis of the HSPCs and immune cells was performed. The full blood count values in PB were determined using the whole blood by an automatic hematology analyzer (XI-800, Sysmex).

### Peripheral Blood Mononuclear Cell Isolation

Peripheral blood mononuclear cells (PBMCs) were isolated from 5 ml of whole blood by density centrifugation using Lymphoprep™ (catalog no. 07851, STEMCELL Technologies). In brief, peripheral blood was diluted with five volumes of phosphate-buffered saline (PBS) and 2% fetal bovine serum (FBS) (PBS + 2% FBS; Thermo Fisher Scientific). Equal amounts of diluted blood was layered on top of Lymphoprep™ carefully and then centrifuged at 800 *g* for 30 min at room temperature with brake off. The PBMC layer was removed and retained the PBMC layer at the plasma. Then, it was washed and resuspended in PBS and was quantified using an automated cell counter (Countess II, Thermo Fisher Scientific).

### Flow Cytometric Analysis

**FIGURE 1:**
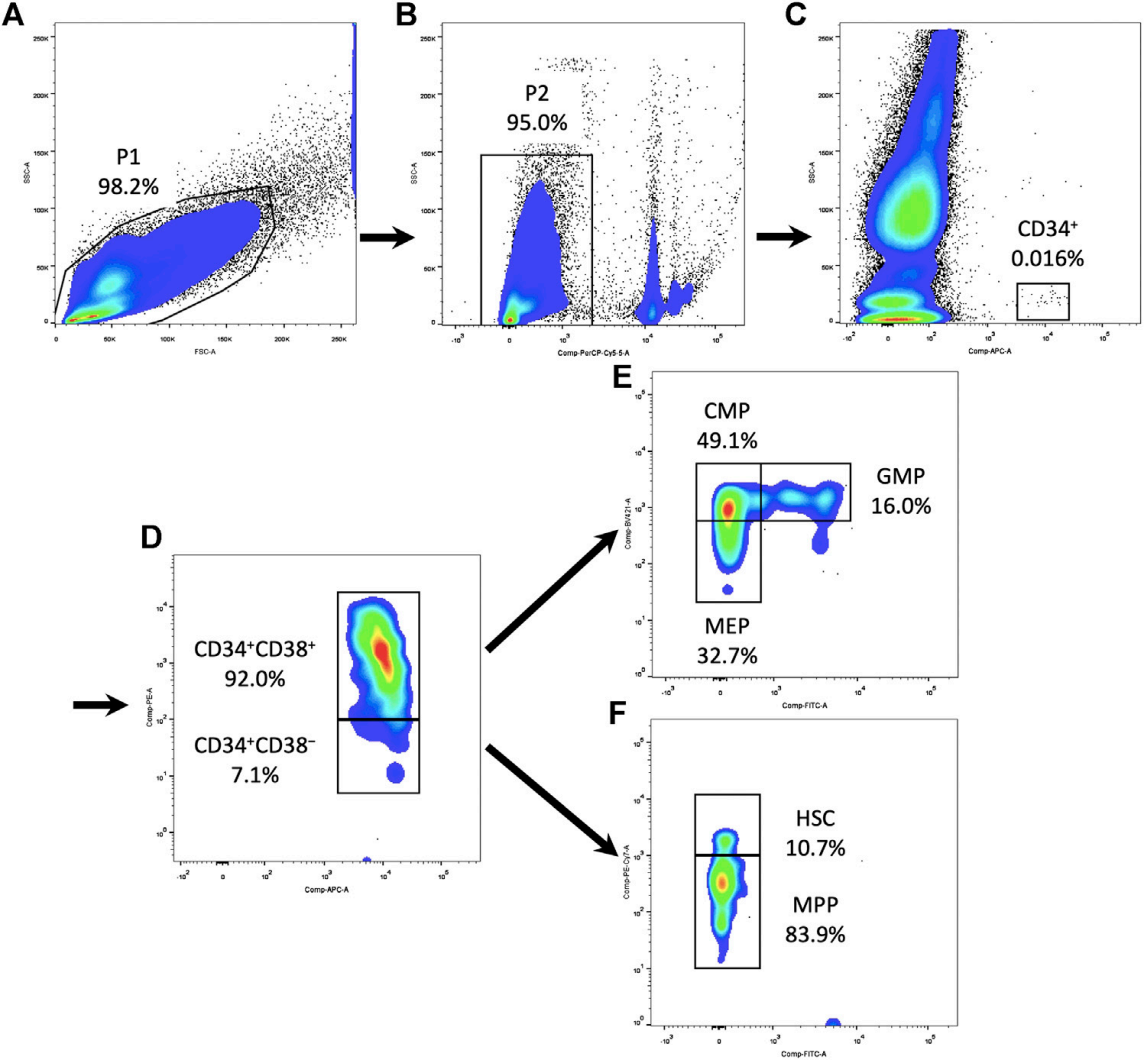
Gating strategy of HSPCs and their subpopulations in the PBMCs. **(A)** Circle all PBMCs and exclude debris (P1). **(B)** Dead cells were excluded using 7-AAD (P2). **(C)** Gating on CD34^+^ cells in SSC-CD34 plot. **(D)** Subdivide CD34^+^ cells into CD34^+^CD38^+^ and CD34^+^CD38^−^ compartments. **(E)** Within the CD34^+^CD38^+^ compartment, CD135 and CD45RA expression defined CMPs, GMPs, and MEPs. **(F)** Within the CD34^+^CD38^−^ compartment, CD90 and CD45RA expression defined HSCs and MPPs. HSPC, hematopoietic stem and progenitor cell; PBMC, peripheral blood mononuclear cell; 7AAD, 7-aminoactinomycin D; SSC, side scatter; CMP, common myeloid progenitor; MEP, megakaryocytic–erythroid precursor; GMP, granulocyte–monocyte progenitor; HSC, hematopoietic stem cell; MPP, multipotential progenitor.

After isolation, fresh PBMCs were resuspended at 10^7^ cells/ml and stained for surface markers in PBS with 2% FBS. A six-color protocol of antibodies (BD Biosciences) was used for the analysis of human HSPCs (van Galen et al., 2014; Notta et al., 2016): APC Mouse Anti-Human CD34 (catalog no. 8334669), PE Mouse Anti-Human CD38 (catalog no. 9154586), FITC Mouse Anti-Human CD45RA (catalog no. 9107502), PE-Cyanine7 Mouse Anti-Human CD90 (catalog no. 9093648), BV421 Mouse Anti-Human CD135 (catalog no. 8201941), and BD Pharmingen™ 7-AAD (catalog no. 51-68981E). After 30 min of incubation, cells were washed three times in PBS before analyses. Stained cells were acquired using a flow cytometer (LSRFortessa, BD Biosciences). At least one million events were recorded in the mononuclear cell gate set on the SSC/FSC morphological plot. The flow cytometric subpopulation was analyzed after gating on CD34^+^ cells in SSC-CD34 plot (Figure 1). For immunophenotypic analysis of immune cells, the following panel of antibodies (BD Biosciences) was used: APC Mouse Anti-Human CD3 (catalog no. 8316946), FITC Mouse Anti-Human CD4 (catalog no. 8037703), PE Mouse Anti-Human CD8 (catalog no. 8299501), and BD Pharmingen™ 7-AAD (catalog no. 51-68981E). Immune cells were gated based on forward/side scatter properties. The flow cytometric subpopulation was further gated on cell surface markers: T-helper cells as CD3^+^CD4^+^ and T-cytotoxic cells as CD3^+^CD8^+^. The results were all analyzed using FlowJo v10 software (Tree Star Inc., Ashland).

### Statistical Analysis

Results were analyzed using GraphPad Prism 7.0 software. Statistical analyses were performed using unpaired, two-tailed, Student’s *t*-test. The correlation study was performed by using the Pearson correlation test. Unless indicated, data were expressed as mean ± SEM. Differences were considered significant when *p* < 0.05, and the following significance levels were used *p < 0.05; **p < 0.01.

## Result

### Phenotypical Changes in HSPCs in Sepsis Patients

**FIGURE 2:**
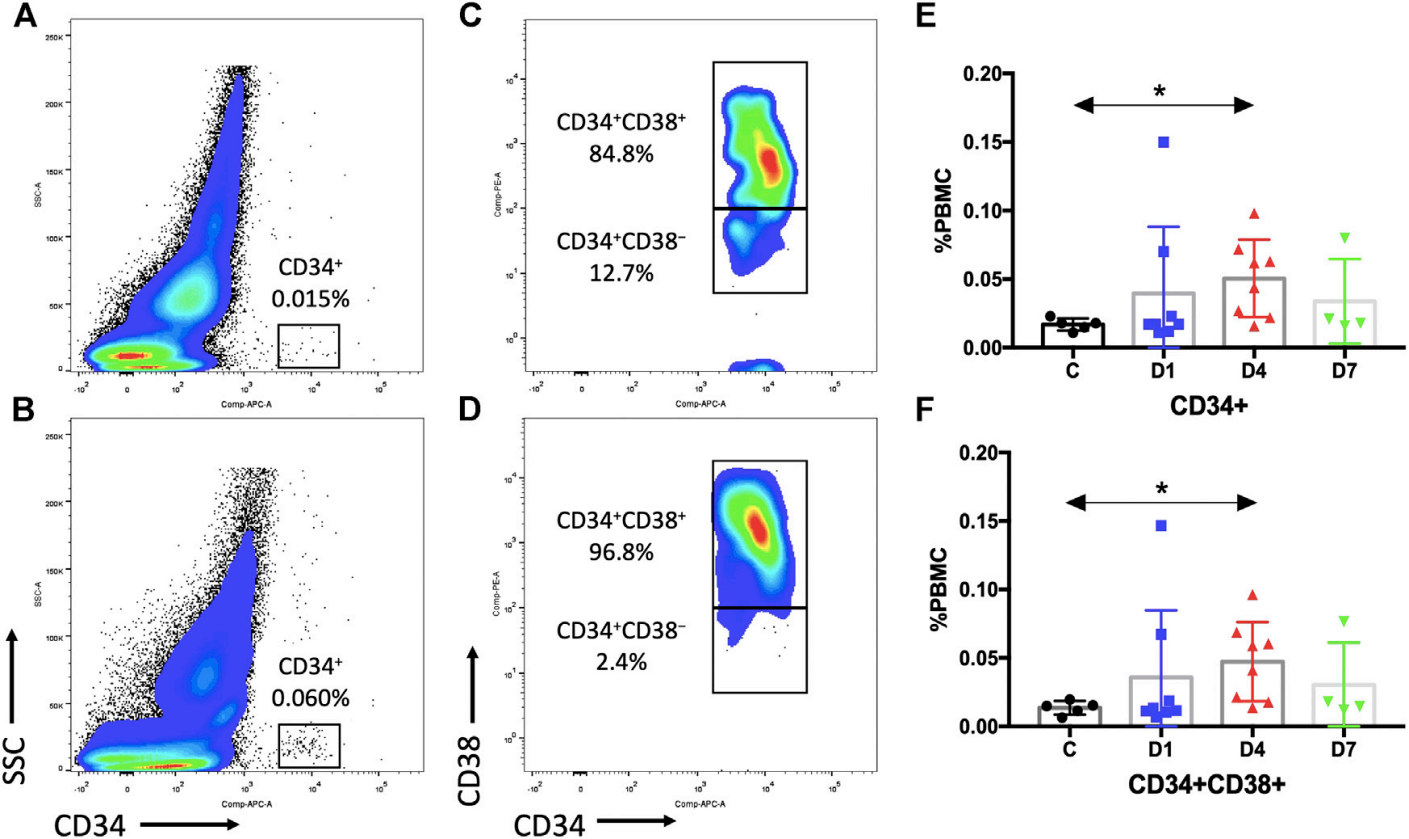
Sepsis patients contain more HSPCs in the PB than healthy control subjects. **(A**,**B)** Representative fluorescence-activated plots of CD34^+^ cells in healthy controls **(A)** and sepsis patients **(B)**. **(C**,**D)** Representative fluorescence-activated plots of CD34^+^CD38^+^ cells in healthy control **(C)** and sepsis patients **(D)**. **(E**,**F)** Kinetic analysis of CD34^+^ cells **(E)** and CD34^+^CD38^+^ cells **(F)**. HSPC, hematopoietic stem and progenitor cell; PB, peripheral blood; PBMC, peripheral blood mononuclear cells; SSC, dide scatter; C, healthy control; D1, sepsis on day 1; D4, sepsis on day 4; D7, sepsis on day 7. (*p < 0.05).

Previous research studies indicated that CD34 is a specific surface marker of human HSPCs (Lynch et al., 2006). We assessed the dynamic changes of CD34^+^ cells in sepsis patients after admission. Results showed that the percentage of PBMCs expressing CD34 in sepsis patients on day 4 was three times higher (0.0505 ± 0.0100%) than that of the healthy control (0.0170 ± 0.0020%) (*p* < 0.05). And it was also increased on days 1 and 7 in sepsis patients, but the difference did not reach significance (Figures 2A,B,E, Supplementary Table S1).

CD34^+^ cells are heterogeneous, which include rare HSCs and hematopoietic progenitor cells (HPCs). We selected CD38 as a surface marker to distinguish between early HSCs (CD34^+^CD38^−^) and HPCs (CD34^+^CD38^+^). The results of the percentage of CD34^+^CD38^+^ cells (day 4: 0.0473 ± 0.0102% vs. 0.0136 ± 0.0022%, *p* < 0.05) were similar as the percentage of CD34^+^ cells (Figures 2C,D,F, Supplementary Table S1). However, no difference in CD34^+^CD38^−^ cells could be observed due to a rare HSC number (Supplementary Table S1).

### Phenotypical Changes in Subpopulation of HSPCs in Sepsis Patients

To precisely identify the changes within the CD34^+^ compartments of human blood that contribute to sepsis, flow cytometric subpopulation analyses were carried out based on immunophenotypic markers. We evaluated the distribution and frequency of immunophenotypic common myeloid progenitors (CMPs; CD34^+^CD38^+^CD135^+^CD45RA^−^), granulocyte–macrophage progenitors (GMPs; CD34^+^CD38^+^CD135^+^CD45RA^+^),megakaryocyte–erythroid progenitors (MEPs; CD34^+^CD38^+^CD135^−^CD45RA^−^), hematopoietic stem cells (HSCs; CD34^+^CD38^−^CD90^+^CD45RA^−^), and multipotential progenitors (MPPs; CD34^+^CD38^−^CD90^−^CD45RA^−^) in sepsis patients and healthy controls (van Galen et al., 2014; Notta et al., 2016).

**FIGURE 3:**
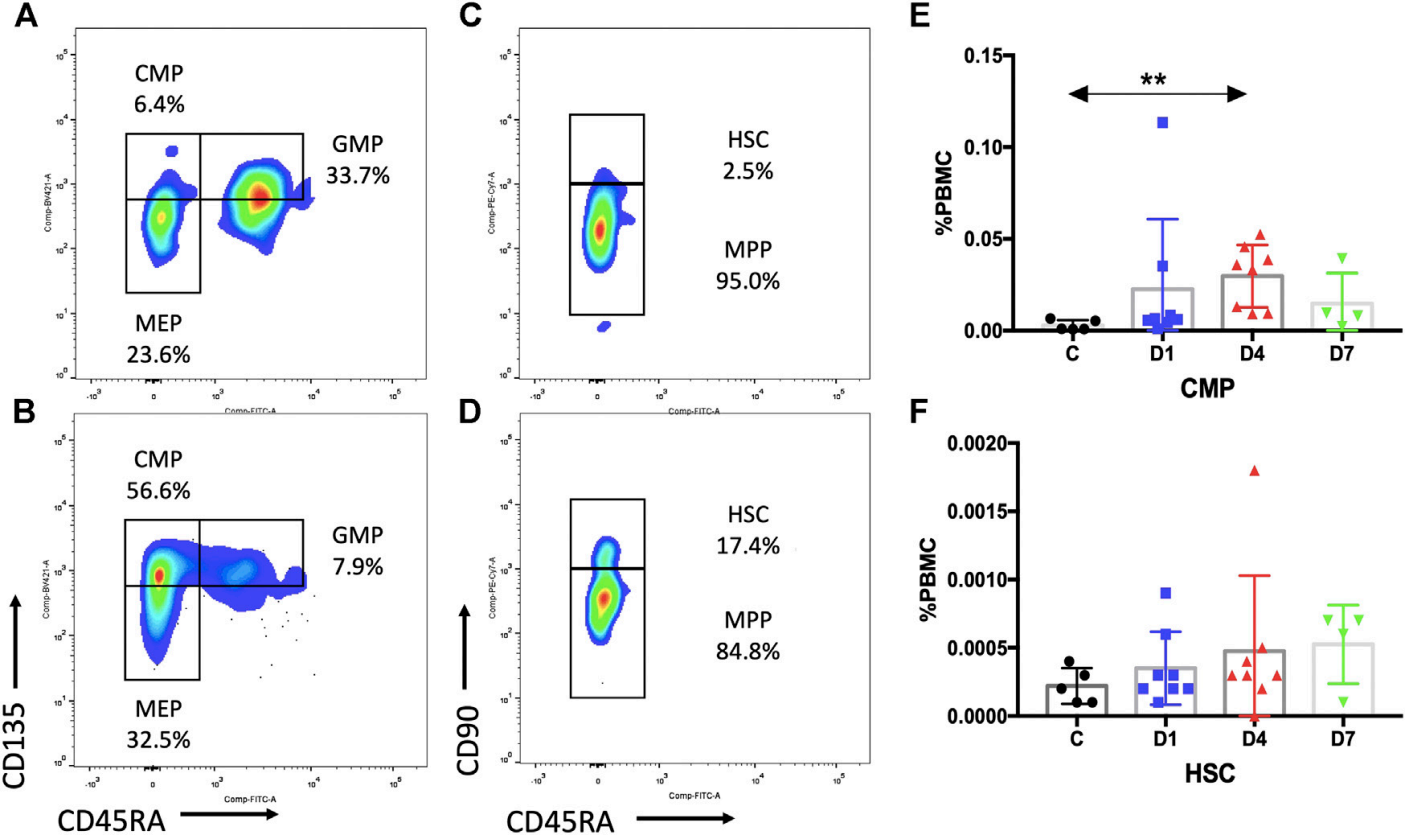
Subpopulation of HSPCs in PB are increased in sepsis patients. **(A**,**B)** Representative fluorescence-activated plots of CMPs, MEPs, and GMPs in healthy control **(A)** and sepsis patients **(B)**. **(C**,**D)** Representative fluorescence-activated plots of HSCs and MPP cells in healthy control **(C)** and sepsis patients **(D)**. **(E**,**F)** Kinetic analysis of CMPs **(E)** and HSCs **(F)**. HSPC, hematopoietic stem and progenitor cell; PB, peripheral blood; CMP, common myeloid progenitor; MEP, megakaryocytic–erythroid precursor; GMP, granulocyte–monocyte progenitor; HSC, hematopoietic stem cells; MPP, multipotential progenitor; PBMC, peripheral blood mononuclear cells; C, healthy control; D1, sepsis on day 1; D4, sepsis on day 4; D7, sepsis on day 7. (**p < 0.01).

**FIGURE 4:**
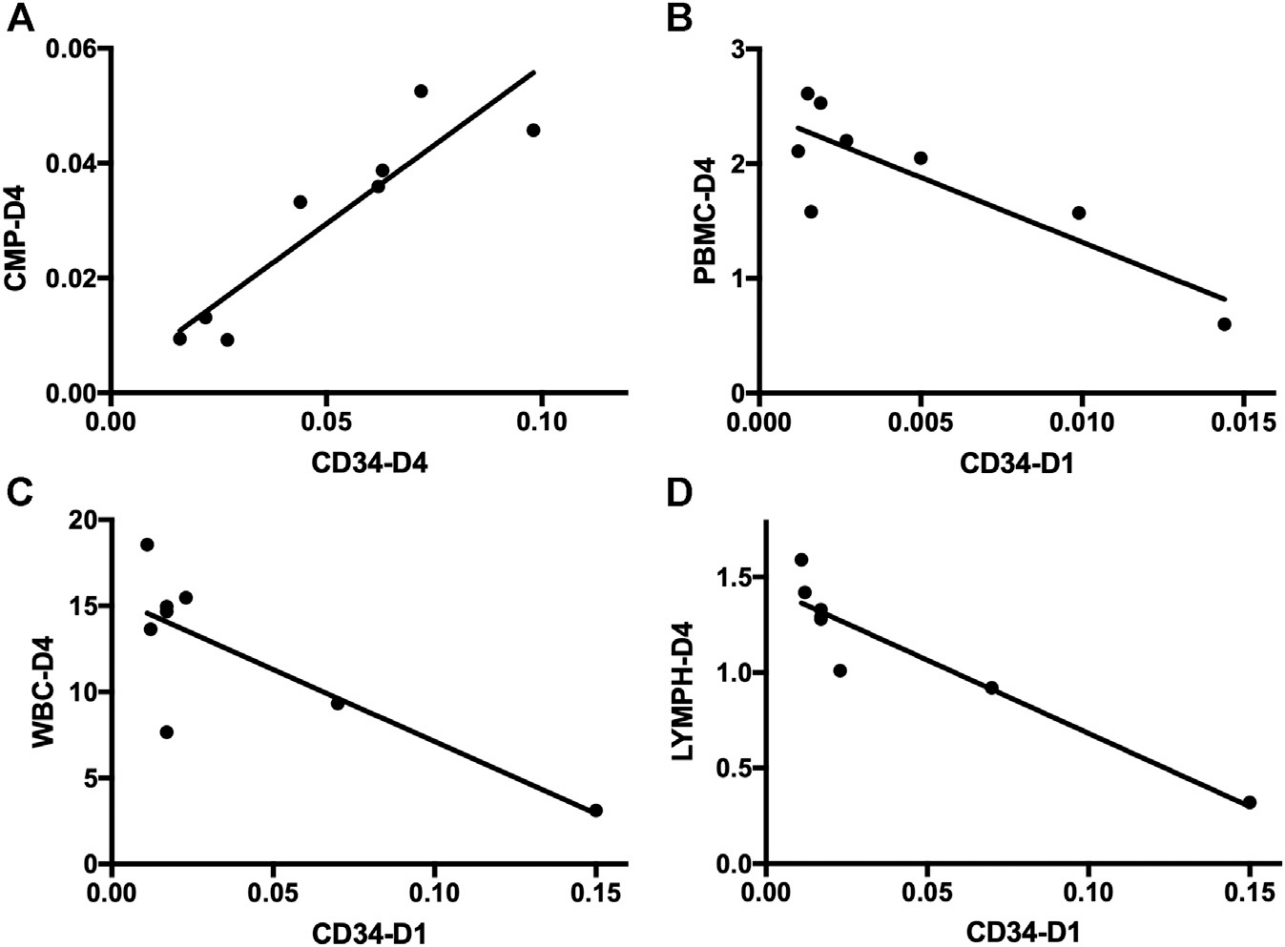
Correlation analysis of HSPCs and immune cells in sepsis patients. **(A)** Correlation analysis of the frequency of CD34^+^ cells on day 4 and CMPs on day 4 in sepsis patients. **(B)** Correlation analysis of the frequency of CD34^+^ cells on day 1 and the absolute number of PBMCs on day 4 in sepsis patients. **(C)** Correlation analysis of the frequency of CD34^+^ cells on day 1 and the absolute number of WBCs on day 4 in sepsis patients. **(D)** Correlation analysis of the frequency of CD34^+^ cells on day 1 and the absolute number of lymphocytes on day 4 in sepsis patients. HSPC, hematopoietic stem and progenitor cell; CMP, common myeloid progenitor; PBMC, peripheral blood mononuclear cell; WBC, white blood cell; LYMPH, lymphocyte.

Subsequent flow cytometric analysis demonstrated that the frequency of CMPs in sepsis patients was increased, and the frequency of CMPs in sepsis patients on day 4 was 10 times higher (0.0297 ± 0.0060%) than the healthy controls (0.0029 ± 0.0013%) (*p* < 0.01) (Figures 3A,B,E, Supplementary Table S1). The MEPs and HSCs in sepsis patients were also increased in frequency and absolute counts, but no statistically significant difference, as compared with the healthy controls (Supplementary Table S1). No obvious difference of GMPs and MPPs could be observed between sepsis patients and healthy controls (Supplementary Table S1). In addition, positive correlations were found between the frequency of CD34^+^ cells and CMPs on day 1 (rs: +0.9916, *p* < 0.01), on day 4 (rs: +0.9124, *p* < 0.01) (Figure 4A), and on day 7 (rs: +0.9834, *p* < 0.05).

### Kinetics of PBMCs and Their Correlation With HSPCs in Sepsis Patients

**FIGURE 5:**
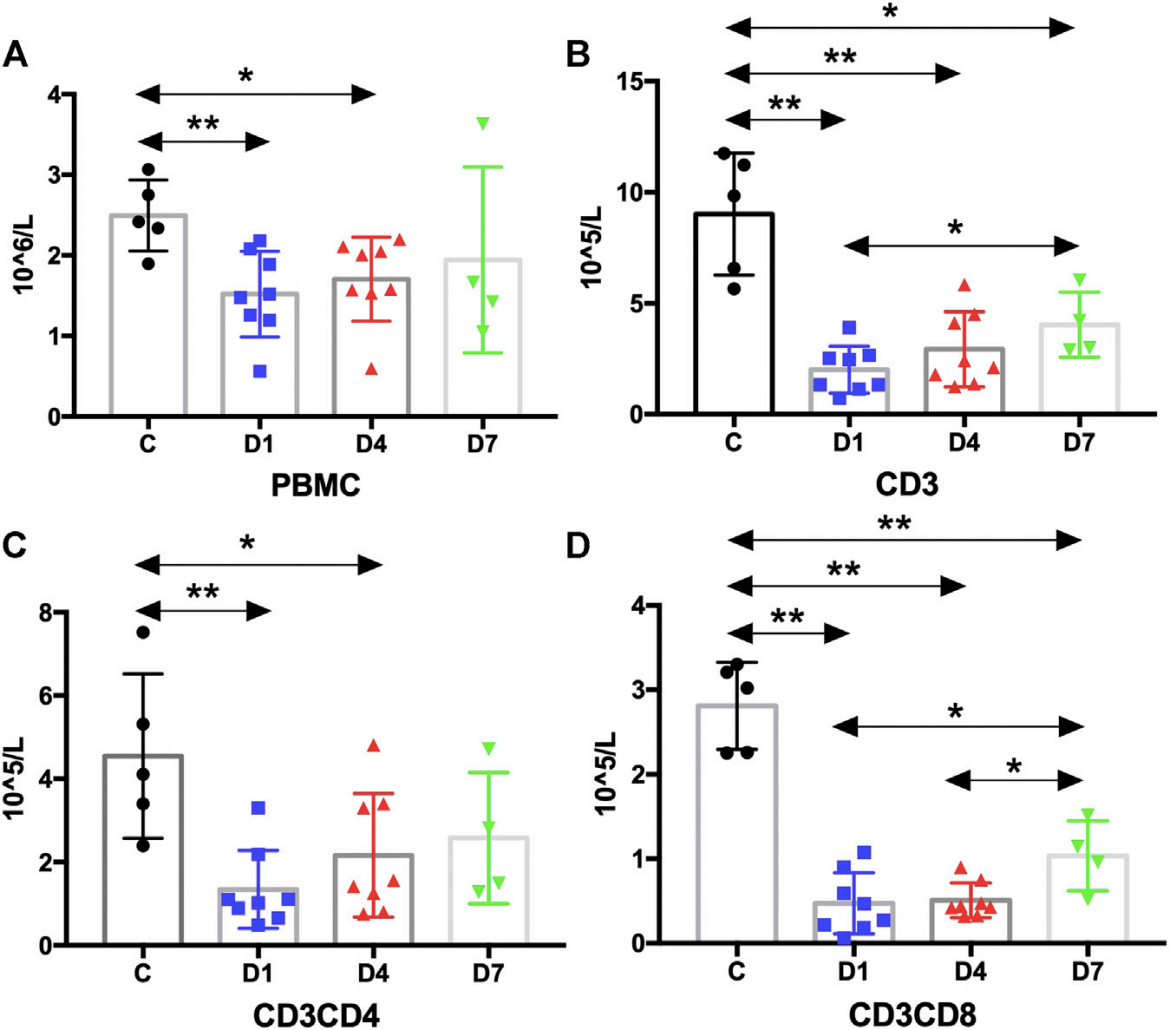
Kinetic analysis of PBMCs and T cells. **(A)** Kinetic analysis of PBMC; **(B–D)** Kinetic analysis of T cells: **(B)** is CD3 T cells, **(C)** is CD3CD4 T cells and **(D)** is CD3CD8 T cells. PBMC, peripheral blood mononuclear cell; C, healthy control; D1, sepsis on day 1; D4, sepsis on day 4; D7, sepsis on day 7. (*p < 0.05, **p < 0.01).

According to the above results, HSPCs expanded during sepsis. Given their fundamental roles in immunity, HSPCs are highly responsive to infections to increase the output of immune cells. It is important to identify whether the HSPC expansion in PB correlates with the number of immune cells. Thus, we measured the absolute counts of PBMCs and T cells and analyzed their correlation with HSPCs in sepsis patients. The results suggest that the number of PBMCs was significantly reduced in sepsis patients on day 1 and on day 4 compared with the healthy controls (*p* < 0.01 and *p* < 0.05, respectively) (Figure 5).

PBMCs are critical components of the immune system and mainly include lymphocytes (T cells and B cells) and monocytes. Next, we analyzed the kinetics of T cells and their subpopulations by flow cytometric analysis. As shown in Figure 5, the number of CD3^+^ T cells and their subpopulations CD3^+^CD4^+^ T cells and CD3^+^CD8^+^ T cells were all significantly decreased compared with the healthy controls. Correlation analysis indicated that there was no relationship between the frequency of CD34^+^ cells on day 4 and the PBMC counts on day 4 (data not shown). But, negative correlations were found between CD34^+^ cells on day 1 and PBMCs on day 4 (rs: −0.8963, *p* < 0.01) (Figure 4B).

### Kinetics of Blood Count and Its Correlation With HSPCs in Sepsis Patients

**FIGURE 6:**
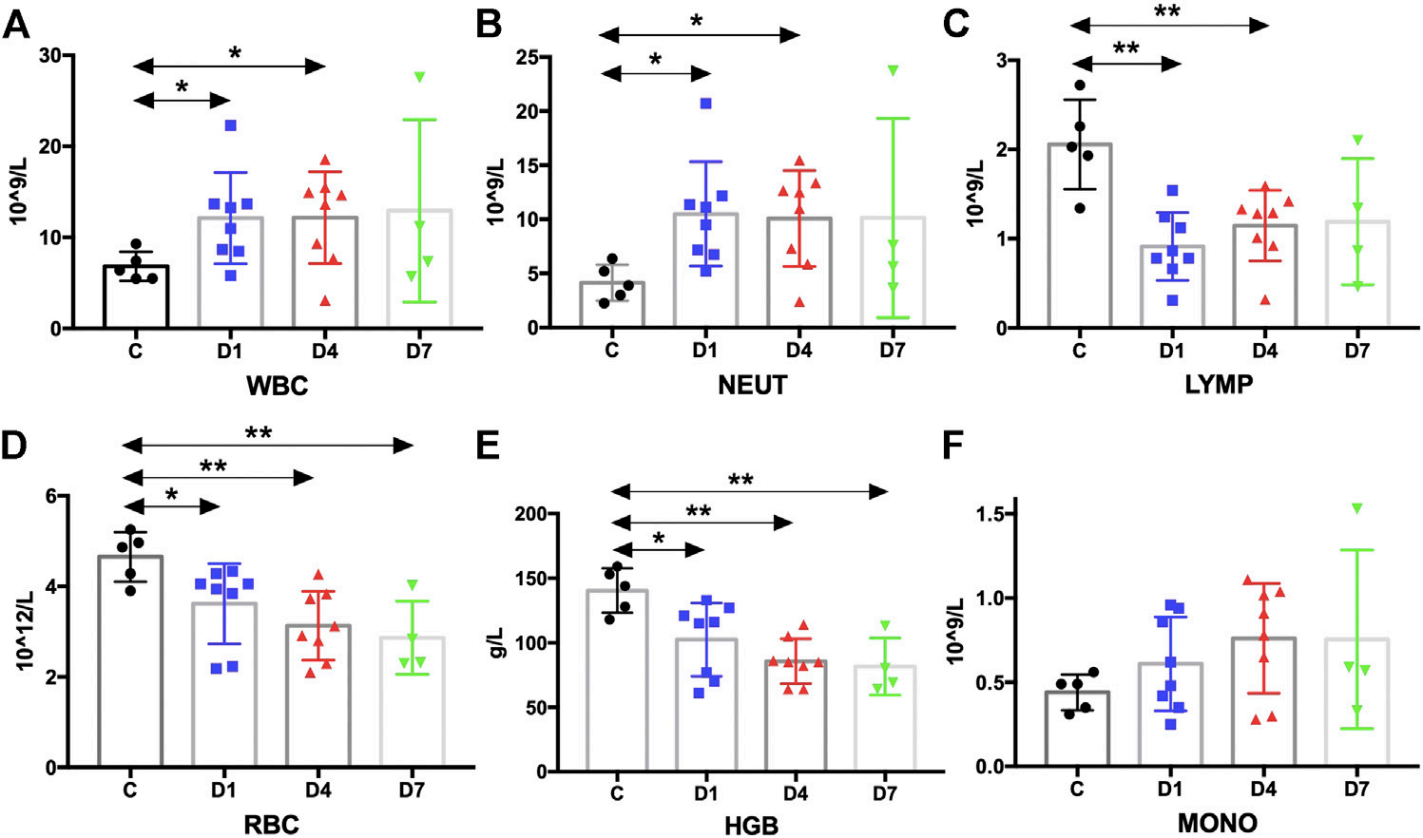
Kinetic analysis of complete blood cell count. **(A–F)** Kinetic analysis of **(A)** white blood cell (WBC); **(B)** neutrophil (NEUT); **(C)** lymphocyte (LYMP); **(D)** red blood cell (RBC); **(E)** hemoglobin concentration (HGB); **(F)** monocyte (MONO); C, healthy control; D1, sepsis on day 1; D4, sepsis on day 4; D7, sepsis on day 7. (*p < 0.05, **p < 0.01).

Complete blood count analysis revealed that the white blood cell (WBC) count was significantly elevated in sepsis on day 1 and on day 4 compared with the healthy controls (*p* < 0.05) (Figure 6A). The same result was observed in neutrophils (Figure 6B). But, compared with the healthy controls, the number of lymphocytes was significantly decreased in sepsis on day 1 and on day 4 (*p* < 0.01) (Figure 6C). Red blood cell (RBC) count and hemoglobin (HGB) concentration were also significantly reduced, whereas no difference in monocytes could be found (Figures 6D–F, Supplementary Table S2). We also aimed to investigate whether the mobilized HSPC correlates with the altered blood count in sepsis. We found that both neutrophil and lymphocyte counts on day 4 in sepsis were negatively associated with the frequency of CD34^+^ cells on day 1 (*p* < 0.05) (Figures 4C,D).

## Discussion

Sepsis is a heterogeneous syndrome associated with acute organ dysfunction and a high risk of death. Due to its heterogeneous, one-size-fits-all approach is one of the barriers to effective therapy (Bhavani et al., 2019). The immune response of sepsis is complex and varies over time (Venet and Monneret, 2018). Therefore, novel methods of early identifying immune status of septic patients could lead to personalized management. The immune cells are derived from HSPC which can respond to many infections. The present study demonstrating that the HSPC and its subpopulation in peripheral blood in sepsis were expansion. Importantly, the changes of HSPC at early timepoints in sepsis have negative correlations with later immune cells. Our results may provide a novel diagnostic indicator and a new therapeutic approach.

Under normal conditions, HSPCs are primarily located in the bone marrow (BM), but a small percentage of HSPCs are migrated into the PB to reconstitute the hematopoietic system under specific stress (Bae et al., 2019). Due to rare HSPC populations, at least one million cells were recorded in order to accurate measurement. In the present study, PB was collected from eight sepsis patients and five healthy controls. In the cases we included, the main site of infection that leads to sepsis is the lung (50%), which is consistent with previous studies (Cecconi et al., 2018). The healthy controls we included were age-matched with the sepsis patients on account that HSCs would increase in frequency with age (Pang et al., 2011).

In the present study, we attempt to identify the changes of HSPCs and their subpopulations circulating in blood of sepsis patients. We found that HSPCs (CD34^+^ cells and CD34^+^CD38^+^ cells) were migrated to the peripheral blood during sepsis, which is consistent with previous studies (Tsaganos et al., 2006; Skirecki et al., 2019). And both the studies indicated that patients with less HSPC counts are accompanied by a greater probability of survival. Sepsis is a result of a systemic response to severe infection, during which mature immune cells were exhausted and needed to be regenerated from the pools of upstream HSPCs. Our previous study found that HSC homeostasis was altered and resulted in the immature immune cells increase in sepsis mice (Wang et al., 2018).

HSPCs (CD34^+^ cells and CD34^+^CD38^+^ cells) are heterogeneous and include many subpopulations which have different functions. HSCs, residing at the top of the hematopoietic system, can give rise to multiple types of HPCs, which including MPPs, CMPs, GMPs, and MEPs. The results of subpopulation analysis of our study suggest that the most pronounced changes are the frequency of CMPs, which may induce an increase in immature cells in sepsis. To date, many research studies observed an increased proportion of immature cells are associated with an increased risk of death (Guérin et al., 2014; Demaret et al., 2015; Hampson et al., 2017). Previous studies indicated that HSCs were expanded and caused functional impairment in sepsis animal models (Rodriguez et al., 2009; Skirecki et al., 2015; Zhang et al., 2016). However, no difference in the immunophenotypically defined HSCs in sepsis were observed due to a rare HSC number in PB.

Mature immune cells have a finite life span and must be continuously replenished from a rare population of HSPCs throughout the life. But sepsis is a consuming syndrome, which directly or indirectly impairs virtually all types of immune cells and results in compromised host immunity (Hotchkiss et al., 2013; Francois et al., 2018). Therefore, we also observed altered immune cell dynamics and analyzed its correlation with HSPCs in sepsis patients. The results indicated that the number of PBMCs and lymphocytes was decreased, whereas the WBC and neutrophil counts were increased. Interestingly, we found a negative correlation between CD34^+^ on day 1 and WBCs, PBMCs, and lymphocytes on day 4 from correlation analysis. Our results suggest that the changes of HSPCs at early time points may provide information to predict later immune status.

But, our study also has some limitations as follows: First, although there is a statistical difference of HSPCs between sepsis patients and healthy controls, the sample size (eight septic patients and five healthy controls) is small. Second, due to the rare HSPC number in peripheral blood, we just investigated its kinetics and correlations with immune cells in sepsis patients. Further studies can pay attention to the functions of HSPCs in addition to their numbers.

## Conclusion

In conclusion, we demonstrate that the HSPCs and their subpopulations in peripheral blood in sepsis were expanded. The changes in HSPCs at early time points in sepsis have negative correlations with later immune cells. To the best of our knowledge, the present study was the first to report kinetics of the subpopulation of HSPCs and their contribution to the immune system in septic patients. Our results may provide a novel diagnostic indicator and a new therapeutic approach.

## Data Availability Statement

The original contributions presented in the study are included in the article/Supplementary Material, further inquiries can be directed to the corresponding authors.

## Ethics Statement

The studies involving human participants were reviewed and approved by the Ethics Committee of Dongzhimen Hospital affiliated to Beijing University of Chinese Medicine. The patients/participants provided their written informed consent to participate in this study.

## Author Contributions

J-XW and H-CS contributed to the conception and design of the study. LW and JW enrolled sepsis patients and healthy control subjects. PW and Y-HL performed the flow cytometric analysis. LW and JW conducted the statistical analysis. PW and Y-HL wrote the first draft of the manuscript. J-XW and H-CS contributed to revising and proofreading the manuscript. All authors contributed and approved the submitted version of the manuscript.

## Funding

This work was supported by startup funds from Beijing University of Chinese Medicine to JW (Grant No. 1000041510051).

## Conflict of Interest

The authors declare that the research was conducted in the absence of any commercial or financial relationships that could be construed as a potential conflict of interest.
