# Supplementary material for: Phenotypical Changes of Hematopoietic Stem and Progenitor Cells in Sepsis Patients: Correlation With Immune Status?
Source: Front Pharmacol. 2021 Jan 19;11:640203. doi: 10.3389/fphar.2020.640203 (PMC7850983; doi:10.3389/fphar.2020.640203)
Supplement: Supplementary file 1 [file datasheet1.docx]

Supplementary Material

# Supplementary Tables

Supplementary Table S1 Percentage and total cell count values of HSPC in the sepsis patients

|  |  | Control | Sepsis D1 | *P* vs Control | Sepsis D4 | *P* vs Control | Sepsis D7 | *P* vs Control |
| --- | --- | --- | --- | --- | --- | --- | --- | --- |
| CD34^+^ | cells/ml | 415.20±42.13 | 421.38±96.27 | 0.9624 | 864.38±168.24 | 0.0647 | 566.25±208.70 | 0.4514 |
|  | % (PBMC) | 0.0170±0.0020 | 0.0396±0.0172 | 0.3286 | 0.0505±0.0100 | 0.0253 | 0.0338±0.015 | 0.2627 |
| CD34^+^CD38^+^ | cells/ml | 327.80±47.24 | 362.38±101.91 | 0.804 | 803.38±167.88 | 0.0526 | 512.25±211.43 | 0.3717 |
|  | % (PBMC) | 0.0136±0.0022 | 0.0358±0.0173 | 0.3399 | 0.0473±0.0102 | 0.0273 | 0.0303±0.0155 | 0.2678 |
| CMP | cells/ml | 72.60±33.62 | 204.38±75.13 | 0.2145 | 533.63±123.70 | 0.0155 | 259.25±114.60 | 0.1265 |
|  | % (PBMC) | 0.0029±0.0013 | 0.0226±0.0135 | 0.281 | 0.0297±0.0060 | 0.0054 | 0.0148±0.0083 | 0.1538 |
| MEP | cells/ml | 122.60±18.14 | 109.50±27.28 | 0.7352 | 153.38±26.86 | 0.4264 | 159.75±77.14 | 0.616 |
|  | % (PBMC) | 0.0050±0.0007 | 0.0090±0.0029 | 0.3117 | 0.0109±0.0039 | 0.2639 | 0.0098±0.0056 | 0.3713 |
| CD34^+^CD38^-^ | cells/ml | 89.43±14.68 | 54.90±9.86 | 0.06 | 63.10±7.43 | 0.1009 | 52.33±2.99 | 0.0427 |
|  | % (PBMC) | 0.0034±0.0003 | 0.0038±0.0004 | 0.5726 | 0.0031±0.0004 | 0.6756 | 0.0035±0.0010 | 0.9177 |
| HSC | cells/ml | 5.40±1.29 | 4.75±0.75 | 0.6474 | 7.50±3.06 | 0.615 | 10.00±4.20 | 0.2846 |
|  | % (PBMC) | 0.0002±0.0001 | 0.0004±0.0001 | 0.3372 | 0.0005±0.0001 | 0.3411 | 0.0005±0.0001 | 0.0695 |
| MPP | cells/ml | 74.00±16.79 | 51.00±10.98 | 0.2545 | 45.13±7.75 | 0.1043 | 36.25±5.19 | 0.0951 |
|  | % (PBMC) | 0.0028±0.0005 | 0.0033±0.0004 | 0.517 | 0.0023±0.0003 | 0.3408 | 0.0025±0.0008 | 0.7096 |
| PBMC | 10^6cells/ml | 2.50±0.19 | 1.52±0.188 | 0.0058 | 1.91±0.23 | 0.1036 | 1.95±0.58 | 0.3524 |
|  | % (WBC) | 38.42±5.35 | 13.34±1.67 | 0.0002 | 16.48±0.97 | 0.0003 | 22.38±3.71 | 0.0525 |
| WBC | cells/ml | 6.81±0.71 | 12.11±1.77 | 0.045 | 12.17±1.79 | 0.044 | 12.94±5.01 | 0.213 |

HSPC, Hematopoietic stem and progenitor cells; D1, Sepsis on day 1; D4, Sepsis on day 4; D7, Sepsis on day 7; PBMC, Peripheral blood mononuclear cells; CMP, Common myeloid progenitor; MEP, Megakaryocytic-erythroid precursor; HSC, Hematopoietic stem cells; MPP, Multipotential progenitor; WBC, White blood cell.

Supplementary Table S2 Percentage and total cell count values of HSPC in the sepsis patients

|  | Control | Sepsis D1 | *P* vs Control | Sepsis D4 | *P* vs Control | Sepsis D7 | *P* vs Control |
| --- | --- | --- | --- | --- | --- | --- | --- |
| WBC | 6.81±0.71 | 12.11±1.77 | 0.0451 | 12.17±1.79 | 0.044 | 12.94±5.01 | 0.2127 |
| RBC | 4.65±0.25 | 3.62±0.31 | 0.0399 | 3.13±0.27 | 0.0027 | 2.87±0.40 | 0.0055 |
| HGB | 140.40±7.66 | 102.50±10.04 | 0.0217 | 85.63±6.17 | 0.0002 | 81.50±11.02 | 0.0027 |
| PLT | 207.60±10.47 | 172.25±46.17 | 0.5672 | 107.75±39.35 | 0.0773 | 133.00±36.58 | 0.0658 |
| NEUT | 4.14±0.74 | 10.50±1.71 | 0.0171 | 10.07±1.57 | 0.0163 | 10.13±4.60 | 0.1898 |
| LYMPH | 2.06±0.22 | 0.91±0.13 | 0.0007 | 1.15±0.14 | 0.0038 | 1.19±0.35 | 0.0678 |
| MONO | 0.44±0.05 | 0.61±0.10 | 0.2238 | 0.76±0.12 | 0.0593 | 0.76±0.27 | 0.2287 |
| NEUT % | 59.18±5.20 | 85.88±1.67 | 0.0001 | 81.61±1.33 | 0.0003 | 73.70±4.95 | 0.088 |
| LYMPH % | 31.90±5.44 | 8.28±1.56 | 0.0003 | 10.03±1.05 | 0.0004 | 16.25±3.89 | 0.062 |
| MONO % | 6.52±0.48 | 5.06±0.50 | 0.0736 | 6.45±0.57 | 0.9338 | 6.13±0.57 | 0.6075 |

D1, Sepsis on day 1; D4, Sepsis on day 4; D7, Sepsis on day 7; WBC, White blood cell; RBC, Red blood cell count; HGB, Hemoglobin concentration; PLT, Platelet count; NEUT, Neutrophils; LYMPH, Lymphocytes; MONO, Monocytes.
