# Supplementary figures and images for: Phenotypical Changes of Hematopoietic Stem and Progenitor Cells in Sepsis Patients: Correlation With Immune Status?
Source: Front Pharmacol. 2021 Jan 19;11:640203. doi: 10.3389/fphar.2020.640203 (PMC7850983; doi:10.3389/fphar.2020.640203)

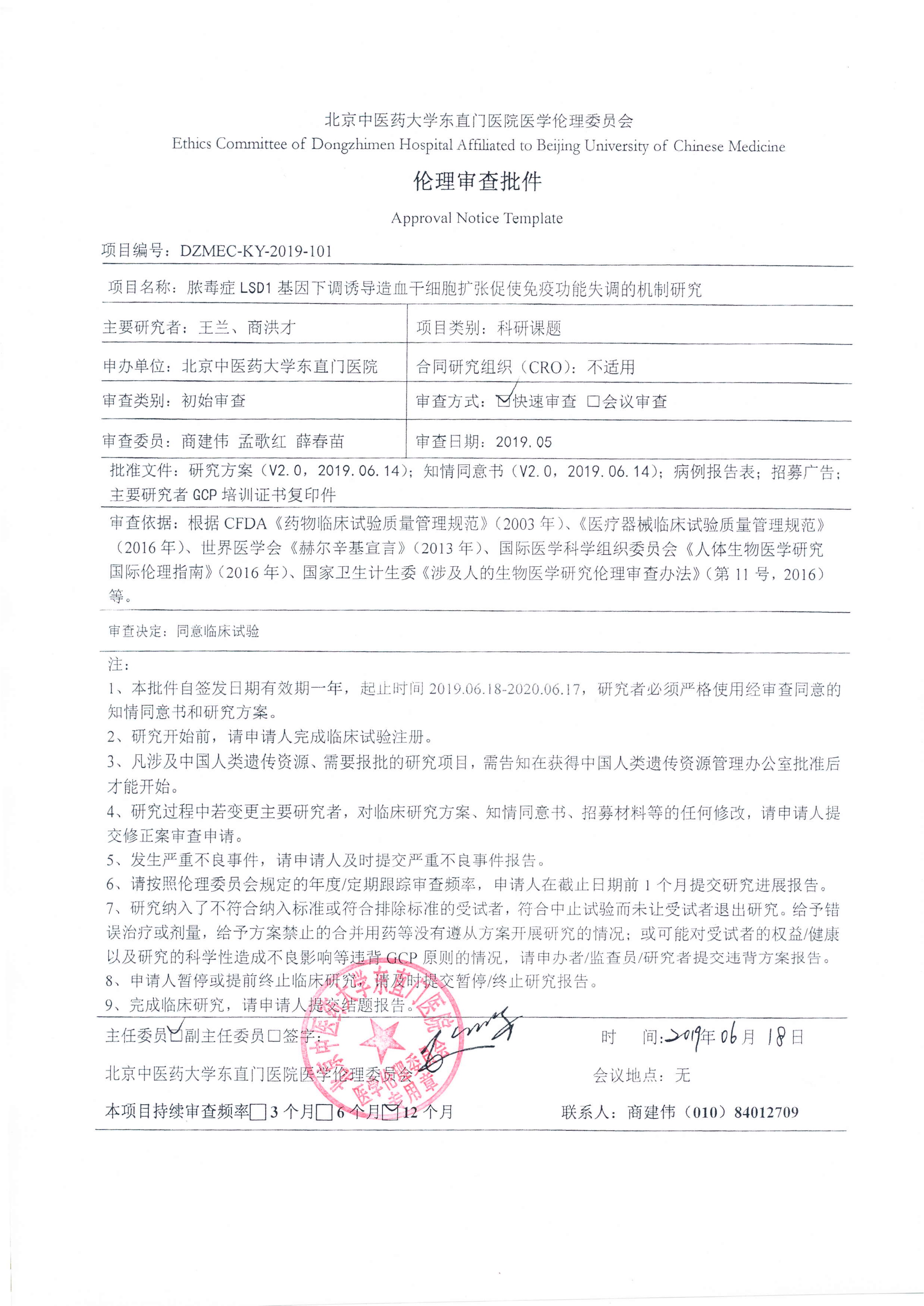

Supplement: Supplementary file 2 [file image1.jpg]
